# Proteomic Profiling of Early Chronic Pulmonary Hypertension: Evidence for Both Adaptive and Maladaptive Pathology

**DOI:** 10.4172/2161-105X.1000241

**Published:** 2015-01-23

**Authors:** Abdulhameed Aziz, Anson M. Lee, Nneka N. Ufere, Ralph J. Damiano, Reid R Townsend, Marc R. Moon

**Affiliations:** Division of Cardiothoracic Surgery and Department of Medicine, Washington University School of Medicine, Saint Louis, Missouri, USA

**Keywords:** Protein expression, Right heart, Hypertrophy, Pulmonary hypertension, Proteome, Proteomics

## Abstract

**Background:**

The molecular mechanisms governing right atrial (RA) and ventricular (RV) hypertrophy and failure in chronic pulmonary hypertension (CPH) remain unclear. The purpose of this investigation was to characterize RA and RV protein changes in CPH and determine their adaptive versus maladaptive role on hypertrophic development.

**Methods:**

Nine dogs underwent sternotomy and RA injection with 3 mg/kg dehydromonocrotaline (DMCT) to induce CPH (n=5) or sternotomy without DMCT (n=4). At 8-10 weeks, RA and RV proteomic analyses were completed after trypsinization of cut 2-D gel electrophoresis spots and peptide sequencing using mass spectrometry.

**Results:**

In the RV, 13 protein spots were significantly altered with DMCT compared to Sham. Downregulated RV proteins included contractile elements: troponin T and C (-1.6 fold change), myosin regulatory light chain 2 (-1.9), cellular energetics modifier: fatty-acid binding protein (-1.5), and (3) ROS scavenger: superoxide dismutase 1 (-1.7). Conversely, beta-myosin heavy chain was upregulated (+1.7). In the RA, 22 proteins spots were altered including the following downregulated proteins contractile elements: tropomyosin 1 alpha chain (-1.9), cellular energetic proteins: ATP synthase (-1.5), fatty-acid binding protein (-2.5), and (3) polyubiquitin (-3.5). Crystallin alpha B (hypertrophy inhibitor) was upregulated in both the RV (+2.2) and RA (+2.6).

**Conclusions:**

In early stage hypertrophy there is adaptive upregulation of major RA and RV contractile substituents and attenuation of the hypertrophic response. However, there are multiple indices of maladaptive pathology including considerable cellular stress associated with aberrancy of actin machinery activity, decreased efficiency of energy utilization, and potentially decreased protein quality control.

Chronic pulmonary hypertension (CPH) is a debilitating disorder that remains one of the most vexing, life-threatening clinical problems in cardiopulmonary medicine [1-3]. While CPH is a disease process that often originates in the lungs, it is the downstream consequences that produce right heart failure and death in the majority of patients. As such, understanding the differential cellular response to CPH within the right atrium and ventricle is paramount for developing guided molecular interventions. Such therapy is needed to arrest the negative metabolic perturbations that are the direct consequence of prolonged pressure overload.

Over the past decade, investigators have developed an appreciation of the macroscopic physiologic impact of CPH on right atrial (RA) and ventricular (RV) mechanics [1-3]. Others have cultivated theories of hypertrophic pathophysiology, but the specific putative mechanisms underlying right heart perturbations in CPH remain unclear [1-3]. Classically, studies evaluating CPH-induced effects on the right ventricle mimic comparable work accomplished on the left side of the heart and are, therefore, centered on protein expression changes in calciumdependent pathways using small animal models [4-7]. Although these studies have improved our understanding of calcium regulation, there exists a vacuum in delineating the molecular mechanisms responsible for RA and RV adaptation in CPH and the ultimate degenerative modulation that produces hypertrophy and failure.

Growing evidence suggests the contribution of contractile machinery, reactive oxygen species production, and energy metabolism in the early RV hypertrophic process [8-10]. Significant improvements in mass spectrometry, bioinformatics technology, and computer software now make a proteomic-based approach possible to identify the cellular and molecular changes that occur during various cardiopulmonary disease states such as CPH [11-13]. In the current investigation, changes in protein expression were delineated using proteomic techniques in a well-established canine CPH model [8- 10], the specific aims of which included: 1.) Identify novel mediators of afterload-induced changes in both the right atrium and ventricle, and 2.) Elucidate whether these early changes are likely adaptive or maladaptive to right heart function.

## Methods

All animals received humane care in compliance with the “Principles of Laboratory Animal Care” formulated by the National Society for Medical Research and the “Guide for the Care and Use of Laboratory Animals” prepared by the National Academy of Sciences and published by the National Institutes of Health. The study was approved by the Washington University School of Medicine Animal Studies Committee and conducted according to Washington University policy.

### Dehydromonocrotaline synthesis

Dehydromonocrotaline (DMCT), a well-known pulmonary endothelial toxin known to cause progressive pulmonary injury, vascular remodeling, and smooth muscle hypertrophy, was used to induce CPH. Commercially available monocrotaline (Sigma-Aldrich, St. Louis, MO) was converted to toxic, bioactive DMCT as previously described (canines lack liver cytochrome oxidase to convert monocrotaline to the toxic form) [14]. Purity of product was confirmed using nuclear magnetic resonance analysis.

### Initial surgical preparation

Nine adult dogs (20-25 kg) were anesthetized with propofol (5-7 mg/kg) and intubated and ventilated using isoflurane (1.5-2.5%, tidal volume 10 mL/kg) after a 7-10 day acclimation period to our large animal facility. Animals were monitored continuously throughout the procedure with pulse oximetry, surface ECG, body temperature (maintained using a table warmer), and frequent arterial blood gases with supplemental oxygen and sodium bicarbonate to maintain a normal acid-base balance and arterial oxygen tension. Micromanometer-tipped pressure catheters (Millar Instruments, Inc., Houston, TX) were zeroed in a 25°C water bath for 30 minutes prior to insertion.

A median sternotomy was performed, leaving the pericardium intact, except for small incisions to permit instrumentation of the heart. A 1-cm incision was made in the pericardium over the anterior RV free wall, and a 5-Fr pressure catheter (MPC-500) was introduced through a pursestring suture. Baseline RV pressure (RVP) was recorded during steady-state conditions in duplicate during suspended ventilation. The micromanometer was replaced with an indwelling 5-Fr fluid-filled pressure catheter (Access Technologies, Skokie, IL) that was tunneled through the right lateral chest wall and connected to a small reservoir covered with a silicone membrane for weekly pressure monitoring throughout the remainder of the study.

Prior to chest closure, five animals underwent RA infusion of DMCT (prepared within 24 hours of use) at 3 mg/kg dissolved in 0.1 mL/kg of dimethylformamide (solvent) to produce pulmonary injury and CPH (DMCT group) [15]. Four animals underwent the identical surgical preparation, but without DMCT infusion (Sham group). Postoperatively, standard antibiotic prophylaxis was continued for 5 days, and furosemide (10 mg/kg twice daily) was given for 48 hours to reduce the incidence of DMCT-induced pulmonary edema, a typical cause of early death in dogs following infusion of pure agent if not treated aggressively [15].

### Subsequent data acquisition and tissue extraction

Animals underwent weekly interrogation of the RVP catheter while they were comfortable and at rest. Unpredictably throughout the experiment, intermittent or permanent catheter thrombosis precluded adequate RVP measurement in some dogs. Only values with an excellent wave form and stable RVP were included in the final data analysis and statistically evaluated. After 8-10 weeks, animals were anesthetized as described above and underwent redo sternotomy. For consistency, a micromanometer-tipped catheter was again used to measure RVP intraoperatively under general anesthesia. The azygous vein and vena cava were ligated, and the right inferior pulmonary vein and inferior vena cava were expeditiously vented before aortic crossclamping and infusion of 400 mL Lactated Ringer’s into the aortic root to arrest the heart. Frozen Lactated Ringer’s slush was placed in the chest to further expedite arrest and tissue preservation. Following arrest, 100 mL of Lactated Ringer’s with 1 mL protease inhibitor cocktail (Sigma-Aldrich, St. Louis, MO) was infused for protein preservation. The heart was excised, the right atrium and ventricle were each divided into 5 horizontal sections and cut into 1 cm vertical intervals, then snap frozen in liquid nitrogen and stored at -80°C.

### Sample preparation

Although proteomic analysis has not previously been performed simultaneously for the right atrium and ventricle, the techniques used in this study were similar to those previously published by our group for other tissues [16]. Briefly, RA and RV specimens (approximately 1 cm^2^) were covered with liquid nitrogen in a pre-cooled Biopulverizer (Biospec Products, Inc.), crushed to a fine powder, and solubilized in lysis buffer. Total protein content for each animal was determined using the Advanced Protein Assay (Cytoskeleton, Inc.), and equal protein samples were used from each animal to form pools of RA and RV tissue for the Sham and DMCT groups. A total pool was generated using equal amounts of each pool to represent all proteins found in the study. An aliquot containing 50 μg of protein from each sample was diluted to 50μL with lysis buffer and labeled with charge-matched cyanine dyes (Sham - Cy2, DMCT - Cy5). The total pool sample was labeled using Cy3 followed by labeling reactions.

### Two-Dimensional difference in-gel (2D-DIGE)

2D-DIGE and image analysis was conducted as previously described [16]. The DeCyder (v. 6.5) difference in-gel analysis module was used to identify gel feature boundaries and calculate abundance ratios using a normalization algorithm. Standard parameters were used to determine boundaries estimating 10,000 spots per image. Gel artifacts were removed by software from each gel image using a peak volume filter set at 10,000. Additional gel artifacts (*e.g*. water spots, dust particles) were excluded manually. A set of 24 gel features (protein spots) were selected from each gel for spot volume ratios exceeding a two standard deviation threshold. Ultimately, these spots were chosen on the basis of a fold difference change greater than 1.5 (increase or decrease in DMCT relative to Sham) and quality of 3-D spot image rendering. Spots were excised robotically (ProPic, Genomic Solutions) using a triangulation algorithm adapted with in-house software. The gel pieces were digested in situ with trypsin.

### Mass spectrometry analysis

Samples were processed and analyzed using nano-reversedphase high performance liquid chromatography interfaced to an electrospray-linear ion trap-Fourier transform ion cyclotron mass spectrometer (LTQ-FT, Thermo-Finnigan) as previously described.(16) The retrieved data were processed using MASCOT Distiller, version 2.1.1.0 (Matrix Science, Oxford, U.K.) and searched using MASCOT version 2.2.04 against the 20,080,708 non-redundant Genbank protein database, restricting output to mammals. The resulting data files were imported into Scaffold, ver. 2.02.03 (Proteome Software, Portland, OR) to identify proteins with greater than or equal to 95% confidence and to determine the spectral counts for each protein. Of the 24 spots chosen in both groups for mass spectrometry, only those meeting specific threshold criteria in Scaffold (99% minimum protein, at least 2 unique peptides, and 95% minimum peptide length) were further analyzed. The majority of selected spots reflected more than one protein. Only the two highest proteins, based on unique spectral count, were included in the final data interpretation. Unique spectral counts were used to identify likelihood of protein dominance within a specific protein spot. Differences in protein expression between the Sham and CPH groups were identified based on their respective cyanine dye label (Sham - Cy2, DMCT - Cy5).

### Statistical analysis

Hemodynamic data obtained during steady-state recording are reported as mean ± standard deviation. Data obtained during baseline and at 8-10 weeks were compared using paired t-test. Differences were considered significant at a level of p<0.05. For protein analysis, Scaffold Software was designed to identify proteins with greater than or equal to 95% confidence with the following specific threshold criteria: 99% minimum protein, at least 2 unique peptides, and 95% minimum peptide length.

## Results

### Development of CPH with DMCT

**Figure 1:**
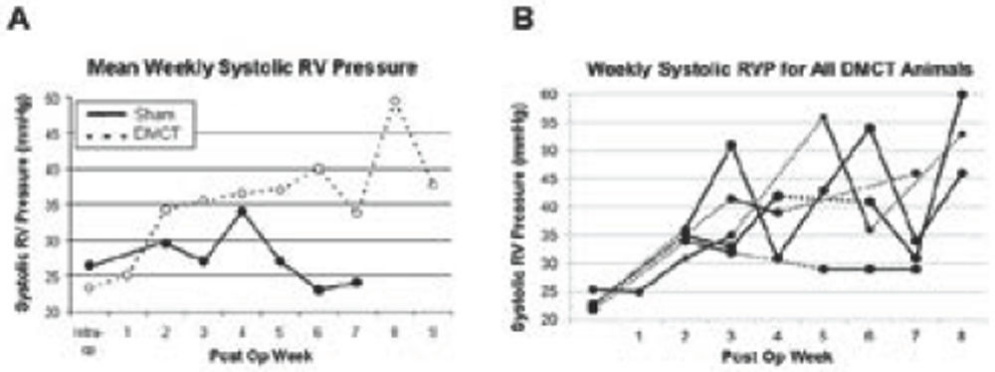
A) Average weekly systolic right ventricular (RV) pressure measurements in the Sham group (solid line, closed circles) and dehydromonocrotaline (DMCT) group (dotted line, open circles). B) Weekly interrogation of right ventricular pressure (RVP) catheter in all five dehydromonocrotaline (DMCT) dogs. Dashed lines connect successive measurements if greater than one week apart.

For the Sham group, intraoperative systolic RVP (anesthetized) was not different at baseline (26 ± 4 mmHg) compared to the final operation (27 ± 6 mmHg) (p=0.56). For the DMCT group, intraoperative systolic RVP (anesthetized) increased by 36% from 23 ± 1 mmHg at baseline to 31 ± 3 mmHg at the final operation (p=0.03). Figure 1A summarizes the mean weekly systolic RVP (conscious) for the DMCT and Sham groups, demonstrating persistent elevation of RVP beyond week one in the DMCT but not Sham groups. Figure 1B demonstrates RVP measurements for each individual dog in the DMCT group.

### Differential proteomic profiles of right atrium and ventricle

**Figure 2:**
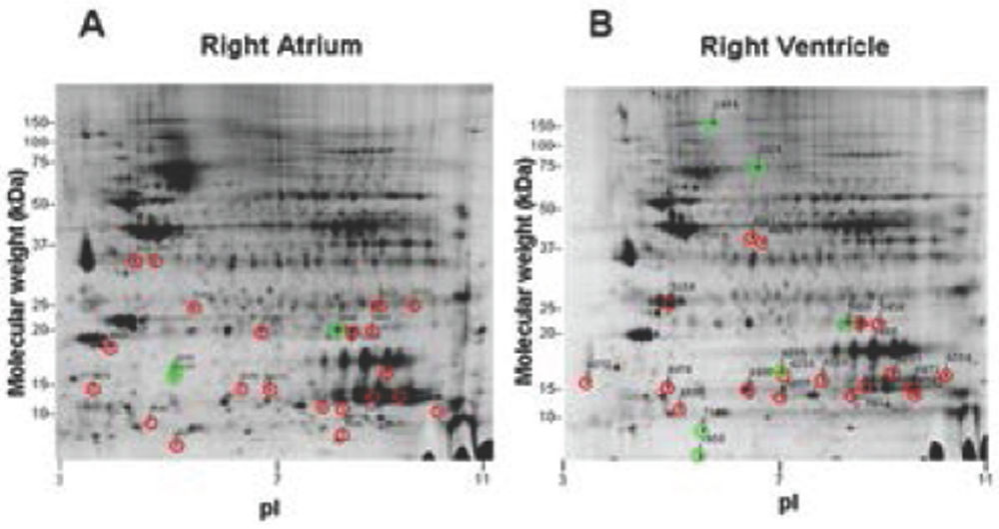
Two-dimensional electrophoresis gels for the right atrium (A) and right ventricle (B). Twenty-four spots were chosen for subsequent mass spectrometry from each gel, identified by a green circle (greater than 1.5 fold increase, DMCT versus Sham) or red circle (greater than 1.5 fold decrease, DMCT versus Sham).

Of 10,000 spots generated by Decyder Software on 2-D gels for the RA and RV samples, over 80% were initially eliminated after establishing a threshold change of 1.5 fold protein quantity or greater (either increase or decrease) for the DMCT relative to Sham group. After manual assessment of 2-D spots and 3-D spot rendering (evaluating protein quality and spot intensity), 24 spots from each of the RA and RV chambers were selected for further mass spectrometry. Figures 2A and 2B are the 2-D electrophoresis gels for the right atrium and ventricle, respectively. The 24 spots chosen for subsequent mass spectrometry are shown for each gel, identified by a green circle (>1.5 increase DMCT versus Sham) or red circle (>1.5 decrease DMCT versus Sham).

After mass spectrometry analysis and database searching, spots not meeting the stringent Scaffold filter threshold criteria identified above were subsequently eliminated from the final analysis. Of the 24 spots selected for mass spectrometry from each chamber, two were eliminated from the right atrium and 11 were eliminated from the right ventricle by the filter thresholds, yielding 22 RA protein spots and 13 RV protein spots that were significantly altered in the DMCT versus Sham group. In the right atrium, 20 spots were downregulated greater than 1.5 fold in the DMCT group, while only two spots were upregulated greater than 1.5 fold. In the right ventricle, nine spots were downregulated greater than 1.5 fold in the DMCT group, while four spots were upregulated greater than 1.5 fold.

### Right atrial protein changes

**Figure 3:**
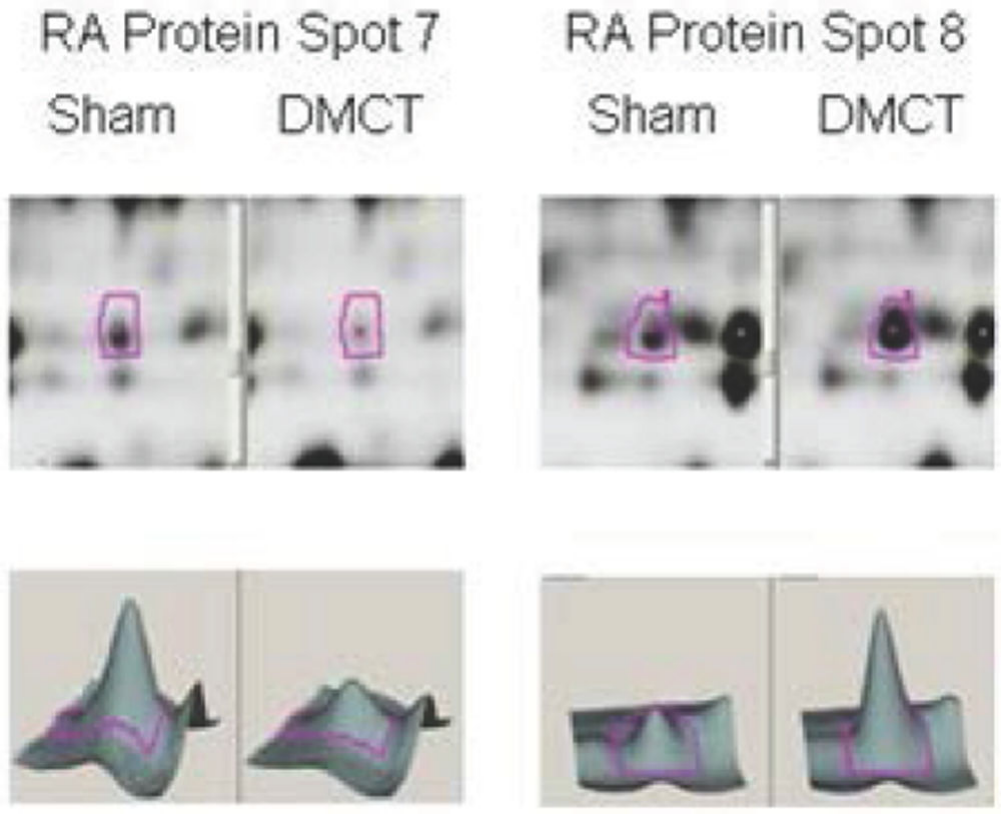
Two-dimensional densitometry (upper panels) of the RA electrophoresis gel and 3-D rendering constructed by Decyder Software (lower panels) for the Sham group (left panels of each spot) versus dehydromonocrotaline (DMCT) group (right panels of each spot). RA protein spot 7: lesser-charged Crystallin alpha B (downregulated-1.7 fold) and RA protein spot 8: highly-charged Crystallin alpha B (upregulated 2.6 fold).

Table 1 summarizes protein identification, quantity change, and the number of unique spectra for each of the RA protein spots with at least 1.5 fold change for the DMCT versus Sham group. In general, there was extensive downregulation of RA proteins involved in contractile function, calcium channel signaling (calmodulin), energy metabolism (Krebs cycle constituents and oxidative phosphorylation chain constituents), ROS scavenging, and ubiquitination. The only RA proteins consistently upregulated for DMCT group were the highlycharged small heat shock protein Crystallin alpha B (associated with inhibition of hypertrophy) and atrial natriuretic factor. Specifically, proteins with the highest unique spectral count per spot that were downregulated in the right atrium for the DMCT group included: Tropomyosin 1 alpha chain (-1.9, spot 2), calmodulin (-2.3, spot 1), slow cardiac myosin regulatory light chain 2 (-2.1, spot 10), atrial/embryonic alkali myosin light chain (-4.4, spot 15) [elements involved in myosin/actin contractile apparatus and calcium signaling], triosephosphate isomerase isoform 1 (-1.8, spot 3), malate dehydrogenase (mitochondrial, -1.8, spot 3), ATP synthase (mitochondrial F0 complex, -1.6, spot 14) [involved in cellular energy production of ATP], lesser-charged Crystallin alpha B isoforms (-1.5 to -1.7, spots 7 and 9) [involved in hypertrophic suppression and protein quality control], periredoxin 5 (-1.8, spot 13) [participator in reactive oxygen species scavenging], fatty acid binding protein (-2.5, spots 16 and 17) [mitochondrial oxidation of fatty acids], “Chain A polylysine induces an antiparallel actin dimmer that nucleates filament assembly” (-2.2, spot 22), and polyubiquitin (-3.5, spot 21) [protein quality control]. The two proteins with high spectral counts that were upregulated in the right atrium for the DMCT group included: highly-charged Crystallin alpha B (2.6, spot 8) [isoform of hypertrophic suppressor, protein quality control] and atrial natriuretic factor precursor (1.68, spot 12) [neurohumoral agent, physiologic diuretic]. Figure 3 demonstrates 2-D densitometry of the RA electrophoresis gel and 3-D rendering for RA proteins: lesser-charged Crystallin alpha B (downregulated, -1.7 fold) and highly-charged Crystallin alpha B (upregulated, 2.6 fold).

### Right ventricular protein changes

**Figure 4:**
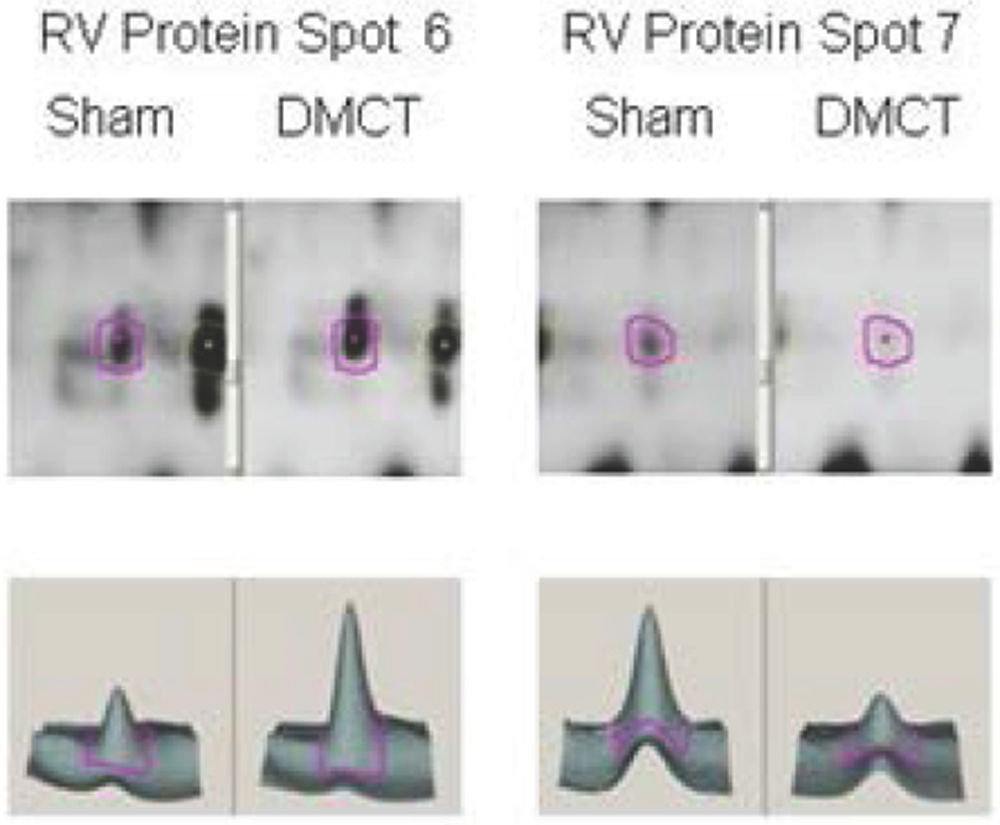
Two-dimensional densitometry (upper panels) of the RV electrophoresis gel and 3-D rendering (lower panels) for the Sham group (left panels of each spot) versus dehydromonocrotaline (DMCT) group (right panels of each spot). RV protein spot 6: highly-charged Crystallin alpha B (upregulated, 2.1 fold) and RV protein spot 7: lesser-charged Crystallin alpha B (downregulated, 2.1 fold).

Table 2 summarizes protein identification, quantity change, and the number of unique spectra for each of the RV protein spots with a 1.5 fold change for the DMCT versus Sham group. In general, there was extensive downregulation of RV proteins involved in contractile function, energy metabolism, and mitochondrial ROS scavenging, but, as was seen in the right atrium, there was upregulation of the highly-charged heat shock protein Crystallin alpha B (inhibition of hypertrophy). Specifically, proteins with the highest unique spectral counts per spot that were downregulated in the right ventricle for the DMCT group included: troponin T isoform (-1.5 to -1.6, spot 3 and 4), myosin light polypeptide (-1.5, spot 5), cardiac ventricular troponin C (-1.5, spot 11), myosin regulatory light chain 2 (MLC-2v) (-1.9, spot 13) [all members of myosin/actin contractile apparatus], isocitrate dehydrogenase (-1.5, spot 4) [cellular energetics – ATP formation], lesser-charged Crystallin alpha B (-1.8 to -2.1, spots 7 and 8), and fattyacid binding protein (-1.5, spot 12), and ROS scavengers superoxide dismutase (cytosolic) and periredoxin 5. Proteins with the highest spectral counts that were upregulated in the right ventricle with CPH included: highly-charged Crystallin alpha B (+2.1, spot 6) [higher charged isoform of hypertrophic suppressor and protein quality control], beta-myosin heavy chain (MHC) (+1.7, spots 1 and 2) [major element of myosin/actin contractile apparatus], and ROS scavengers superoxide dismutase (cytosolic) (+1.8, spot 9) and periredoxin 5 (+1.8, spot 9). Figure 4 demonstrates 2-D densitometry of the RV electrophoresis gel and 3-D rendering for RV proteins: highly-charged Crystallin alpha B (upregulated, 2.1 fold) and lesser-charged Crystallin alpha B (downregulated, -2.1 fold).

## Discussion

Previous investigators vociferate the need for a better understanding of right heart dysfunction in CPH and the detrimental cellular and molecular mechanisms that may be pacified with such knowledge [17,18]. In the current study, a proteomic-based approach was employed in a large animal model that closely recapitulates human hemodynamic changes in CPH to delineate protein changes in both the right atrium and ventricle. Using rigorous threshold filters after mass spectrometry analysis, substantial perturbations were identified in proteins responsible for cellular function and integrity in early hypertrophy. These changes included proteins principally involved in contractile function, energy metabolism, ROS scavenging, suppression of the hypertrophic response, and ubiquitination.

Major components of contractile machinery exhibited significant downregulation in both right heart chambers, with the exception of upregulated beta-MHC in the ventricle. Beta-MHC was upregulated greater than 70% for the DMCT group, and has previously been identified as an early, sensitive marker of hypertrophy in the left ventricle [19]. Beta-MHC is characterized by lower ATP activity, and this reduced consumption can provide greater conservation of energy than alpha-MHC, suggesting an adaptive response to preserving energy [20]. Investigators have also observed that the shift in murine alpha-MHC to beta-MHC may be more than just a marker of disease and may actually contribute to development of the maladaptive state under chronic conditions - manifest as a blunted response to beta-adrenergic stimulation [21]. On the other hand, we show a substantial decline in RV troponin T isoform. Recent work shows that Troponin T mutations exacerbate myofilament calcium sensitivity, but independently contribute to arrhythmia susceptibility [22]. In the right atrium, contributors of actin mechanics were all decreased, including tropomyosin 1 alpha, slow cardiac myosin regulatory light chain, and atrial/embryonic alkali myosin light chain. Reduction in tropomyosin 1 alpha has been associated with dilated cardiomyopathy and familial hypertrophic cardiomyopathy, while slow cardiac myosin regulatory light chain affects appropriate physiologic cardiac sarcomere formation and heart development [23]. The function and significance of atrial alkali myosin light chain’s role in myosin fine-tuning functions remains unclear [24]. These collective RA and RV changes in actin mechanistic proteins imply disadvantageous sarcomere contractility in the chronic state.

Interestingly, the ubiquitously expressed heat shock protein Crystallin alpha B was significantly upregulated in its most highlycharged isoform in both the right atrium and right ventricle for the DMCT group, but downregulated in lesser-charged isoforms. Crystallin alpha B is the most abundant small heat shock protein constitutively expressed in cardiomyocytes, with a major putative role in preventing the stress-induced aggregation of denaturing proteins (protein quality control) and inhibiting the hypertrophic response.(25) Crystallin alpha B has been induced in the left ventricle by pressure overload in a transverse aortic-constriction mouse model, likely by attenuating pressure-overloaded hypertrophic responses and associated NFAT (nuclear factor of activated T-cells) expression in mouse hearts [25]. Therefore, the adaptive effect of Crystallin alpha B upregulation is implicit, especially when considering that Crystallin alpha B functional loss in the pressure-overload left ventricle has been shown to be more pathologic than physiologic. The current findings suggest that the compensatory upregulation and maintenance of protein quality control in the early hypertrophic state is also relevant, not only for the right ventricle, but also for the right atrium with a relatively moderate CPH phenotype (36% rise in systolic RV pressures). This upregulation in protein quality control may be an important compensatory response for the right atrium, as polyubiquitin was significantly downregulated (-3.5 fold), with potential aberration of proteosome degradation of damaged RA proteins.

Proteins involved in cellular energetics and reactive oxygen species regulation were downgraded for the DMCT group in the current investigation. In the atrium, downregulation of multiple members of the Krebs cycle and oxidative phosphorylation chain (including ATP synthase) suggests decreased, or less efficient, ATP generation. Perturbed myocardial energetics are known to occur in the hypertrophic state, and may be reflective of increased tissue acidity or PCO_2_, leading to the classic definition of pathologic hypertrophy as depressed myosin ATPase activity without concordant heart failure [26]. In the current study, manifest changes in mitochondrial proteins integral to cellular energetics were affected, even with only moderate versus severe elevations in pulmonary artery pressures. Furthermore, unopposed formation of reactive oxygen species has been shown to accelerate development of right heart failure in CPH [27], a concept that is supported by associated perturbations in superoxide dismutase and periredoxin 5 in the current report. Decreased fatty-acid binding proteins in both the right atrium and ventricle are consistent with decreased ATP utilization and a transformation from utilization of fatty acid to glucose as a substrate. Lack of myocardial fatty-acid binding protein can lead to an increase of unbound fatty acids and impaired mitochondrial fatty acid oxidation [28]. Collectively, aberration in mitochondrial energetics may be a common thread in exacerbating the untoward responses that contribute to maladaptive homeostatic dysregulation in the right heart with CPH.

**Figure 5:**
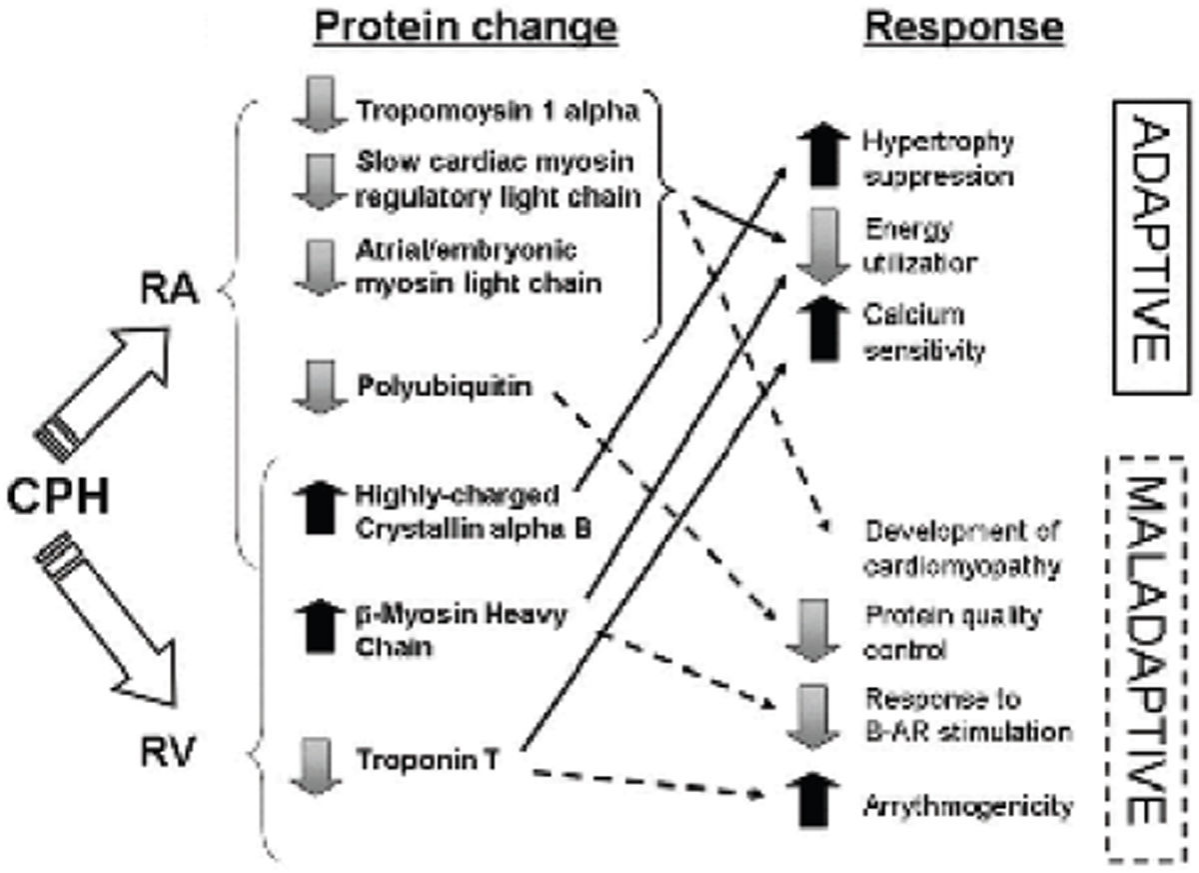
Postulated mechanisms of right atrial (RA) and right ventricular (RV) protein regulation in chronic pulmonary hypertension (CPH) based on the findings of the current proteomic analysis and previously described sequelae of specific protein upregulation or downregulation. Effects are stratified based on their presumed adaptive or maladaptive consequences.

Figure 5 summarizes our postulated adaptive and maladaptive features of early right heart changes in CPH based on the findings of the current proteomic analysis comparing DMCT and Sham groups. Downregulated contractile proteins may initially improve metabolism due to a decrease in energy utilization, but have been shown to play a role in the development of cardiomyopathy. However, though beta-MHC upregulation may also preserve energy utilization, it may impair RV contractility by decreasing the myocardial response to beta-adrenergic receptor stimulation. The resultant hypertrophic stimulation is balanced by highly-charged Crystallin alpha B upregulation (inhibitor of hypertrophy) in both the atrium and ventricle. Downregulation of polyubiquitin is surely maladaptive due to its well-established role in protein quality control. Finally, downregulated RV Troponin T may be both adaptive and maladaptive as it increases calcium sensitivity, while increasing the arrythmogenic potential of the ventricle.

### Potential Limitations

There are several limitations to interpretation of the findings of the current report. Though global protein alterations were compared using the same 2-D gel between groups, individual animal RA and RV tissue were not analyzed for intra-dog comparison, which does not account for baseline genetic variability between the individual animals within each group. In addition, the effect of DMCT varied among administered dogs and induced only a modest rise in RV pressure (36%). Although this was sufficient for analyzing the moderate CPH phenotype, further studies requiring either a prolonged exposure or more substantial degree of RV afterloading will be necessary to investigate the progression from hypertrophy to failure with severe CPH. Other potential limitations include putative toxicity and cell cycle changes in endothelial cells secondary to dehydromonocrotaline [29]. The rigorous threshold filters employed in this study for proteomic analysis facilitated evaluation of substantial protein changes, revealing only the most confident peptide identification data. As a result, these methods were unable to identify other potentially relevant changes, such as unidentified membrane receptor or calcium handling proteins, with important yet less profound quantitative changes.

In summary, the current study evaluated the right heart proteome, both atrial and ventricular, during moderate CPH in dogs. Common themes of decreased ATP synthesis, decreased fatty acid oxidation, and a diminution in many substituents of sarcomere contractile machinery suggest aberrant mitochondrial energetics and decreased contraction efficiency at the expense of a more resilient response to pressure overload (i.e., beta-MHC in the ventricle) and active suppression of the hypertrophic response (Crystallin alpha B in both the atrium and ventricle). Therefore, although classic theories suggest that chronic pressure overload induces an adaptive myocardial response, the current findings, from a more global right heart proteomic evaluation, identify potentially maladaptive traits as well. Further evaluation of the substituents identified in the current report and sequential analysis of more severe phenotypes are warranted to better understand the developmental pathology of the right heart in CPH and to develop molecular therapeutics.

## Figures and Tables

**Table 1:** Right atrial protein changes with chronic pulmonary hypertension identified by mass spectrometry.

Spot	2D Gel ID No.	Protein	GI number	Fold change	#Unique Spectra
1	4480	Tropomyosin 1 alpha chain	gi∣74000377	-2.27	6
		calmodium	gi∣223872 (+10)		6
2	4500	Tropomyosin 1 alpha chain	gi∣74000377	-1.89	9
		sarcolemmal associated protein	gi|790238∣gb|AAA65597.1		2
		atrial/embryonic alkali myosin light chain	gi|508454528∣ref|NP_001002841.1		2
3	5231	Triose-phosphate isomerase isoform 1	gi|40889552|pdb|1R2R|A chain A	-1.8	7
		malate DH, mitochondrial	gi∣73957776		3
4	5257	NipSnap2 protein isoform	gi∣57087439 (+2)	-2	2
		Coiled-coil-helix-coiled-coil-helix domain			
		Containing protein 3 isoform 2	gi∣73975490		2
		Chain A polylysine induces An Antiparellel			
5	5285	Actin Dimer that Nucleates Filament	gi∣20664362	-1.57	2
		atrial/embryonic alkali myosin light chain	gi|508454528∣ref|NP_001002841.1		2
		ATP synthase, H+ transporting,			
6	5585	mitochondrial FO complex, subunit d isoform	gi∣57108097	-1.48	8
		myosin lighy chain 2a	gi∣10864037		3
		myosin heavy chain 6	gi∣127741		3
7	5602	crystalin alpha B	gi∣27805849	-1.77	4
		Transgelin 2	gi∣12803567(+9)		3
		Phosphatidylethanolamine binding protein 1	gi∣114326321		3
		Superoxide dismutase isoform B precursor			
		isoform 3	gi∣73945756(+2)		3
8	5606	crystalin alpha B	gi∣27805849	2.64	6
		Phosphatidylethanolamine binding protein 1	gi∣114326321		3
9	5616	crystalin alpha B	gi∣27805849	-1.54	8
		Superoxide dismutase isoform B precursor			
		isoform 3	gi∣73945756(+2)		6
10	5813	slow cardiac myosin regulatory light chain 2	gi∣50978736	-2.12	14
		atrial/embryonic alkali myosin light chain	gi|508454528∣ref|NP_001002841.1		8
		Apolipoprotein A-I precursor	gi∣73955106		8
11	6133	non-metastatic cells 2, protein	gi∣66864901	-2.14	2
12	6159	Atrial natriuretic factor precursor	gi∣73950787	1.68	6
13	6373	Peroxiredoxin 5	gi∣57099689	-1.75	4
		Superoxide dismutase	gi∣50978674		2
		ATP synthase, H+ transporting,			
14	6390	mitochondrial FO complex, subunit d isoform	gi∣57108097	-1.58	5
		Chian A, polylysine induces an Antiparellel			
		Actin Dimer that Nucleates Filament	gi∣20664362		2
15	6470	atrial/embryonic alkali myosin light chain	gi|508454528∣ref|NP_001002841.1	-4.37	5
		Apolipoprotein A-I precursor	gi∣73955106		3
		mysin light chain 2a	gi∣10864037		3
16	6600	Fatty acid-binding protein, adipocyte	gi∣20138310(+4)	-2.56	3
17	6602	Fatty acid-binding protein, adipocyte	gi∣20138310(+4)	-2.44	2
		Chain A polylysine induces An Antiparellel			
18	6701	Ac tin dimer that Nucleates Filament	gi∣20664362		
		NADH dehydrogenase (ubiquinone) Fe-S	gi∣56711244(+1)		2
19	6718	NADH dehydrogenase (ubiquinone) Fe-S	gi∣56711244(+1)	-2.6	2
		Chain A polylysine induces An Antiparellel			
20	6940	Actin Dimer that Nucleates Filament	gi∣20664362	-1.79	5
		atrial/embryonic alkali myosin light chain	gi|508454528∣ref|NP_001002841.1		2
		desmin	gi∣126138909(+2)		2
21	7146	polubiquitin	gi∣1050930	-3.46	3
		Chian A, polylysine induces an Antiparellel			
22	7267	Actin Dimer that Nucleates Filament	gi∣20664362	-2.2	2
		atrial/embryonic alkali myosin light chain	gi|508454528∣ref|NP_001002841.1		2

**Table 2:** Right ventricular protein changes with chronic pulmonary hypertension identified by mass spectrometry.

Spot	2D Gel ID No.	Protein	GI number	Fold change	#Unique Spectra
1	1491	beta-mysin heavy chain	gi∣12053672	1.77	28
2	2624	beta-mysin heavy chain	gi∣12053672	1.73	13
		Stress-70 protein, mitochondrial precursor	gi∣73969214(+15)		2
3	4031	troponin T isoform 1	gi∣15072321(+7)	-1.64	8
		L-lactate dehydrogenase B chain	gi∣57043188		2
4	4091	troponin T isoform 1	gi∣15072321(+7)	-1.54	5
		isocitrated DH 3 (NAD+) alpha isoform 1	gi∣73951312		4
5	5158	Myosin light polypeptide 3	gi∣57101266	-1.54	20
		Mysin regulatory light chain 2, ventricular/cardiac			
		muscle isoform	gi∣127167(+6)		
6	5450	crystallin, alpha B	gi∣27805849(+1)	2.15	12
		superoxide dismutase [Mn], mitochondrial precursor			
		isoform 4	gi∣73970888(+15)		2
		Myosin light polypeptide 3	gi∣57101266		2
7	5458	crystallin, alpha B	gi∣27805849(+1)	-2.07	5
8	5466	crystallin, alpha B	gi∣27805849(+1)	-1.83	12
		superoxide dismutase [Mn], mitochondrial precursor	gi∣73970888(+15)		7
9	6205	superoxide dismutase 1, soluble	gi∣50978674	1.76	3
		peroxiredoxin 5, mitochondrial precursor	gi∣57099689		3
10	6255	superoxide dismutase 1, soluble	gi∣50978674	1.76	3
11	6372	cardiac ventricular troponin C	gi∣2460249(+3)	-1.56	5
12	6490	Fatty acid-binding protein, heart	gi∣57043188	-1.51	9
		Myosin regulatory light chain 2, ventricular/cardiac			
13	6853	muscle isoform (MLC-2v)	gi∣127167(+6)	-1.89	3
